# Health policy and systems research training: global status and recommendations for action

**DOI:** 10.2471/BLT.15.162818

**Published:** 2016-04-21

**Authors:** Tara M Tancred, Meike Schleiff, David H Peters, Dina Balabanova

**Affiliations:** a London School of Hygiene & Tropical Medicine, Keppel Street, London, WC1E 7HT, England.; b Johns Hopkins Bloomberg School of Public Health, Baltimore, United States of America.

## Abstract

**Objective:**

To investigate the characteristics of health policy and systems research training globally and to identify recommendations for improvement and expansion.

**Methods:**

We identified institutions offering health policy and systems research training worldwide. In 2014, we recruited participants from identified institutions for an online survey on the characteristics of the institutions and the courses given. Survey findings were explored during in-depth interviews with selected key informants.

**Findings:**

The study identified several important gaps in health policy and systems research training. There were few courses in central and eastern Europe, the Middle East, North Africa or Latin America. Most (116/152) courses were instructed in English. Institutional support for courses was often lacking and many institutions lacked the critical mass of trained individuals needed to support doctoral and postdoctoral students. There was little consistency between institutions in definitions of the competencies required for health policy and systems research. Collaboration across disciplines to provide the range of methodological perspectives the subject requires was insufficient. Moreover, the lack of alternatives to on-site teaching may preclude certain student audiences such as policy-makers.

**Conclusion:**

Training in health policy and systems research is important to improve local capacity to conduct quality research in this field. We provide six recommendations to improve the content, accessibility and reach of training. First, create a repository of information on courses. Second, establish networks to support training. Third, define competencies in health policy and systems research. Fourth, encourage multidisciplinary collaboration. Fifth, expand the geographical and language coverage of courses. Finally, consider alternative teaching formats.

## Résumé

**Objectif:**

Examiner les caractéristiques des formations à la recherche sur les politiques et les systèmes de santé dans le monde entier et proposer des recommandations en vue de les améliorer et de les étendre.

**Méthodes:**

Nous avons identifié des institutions qui proposent des formations à la recherche sur les politiques et les systèmes de santé dans le monde entier. En 2014, nous avons recruté des participants au sein des institutions identifiées pour mener une enquête en ligne sur les caractéristiques des institutions et sur les cours dispensés. Les résultats de cette enquête ont été étudiés lors d'entretiens approfondis avec des informateurs clés sélectionnés.

**Résultats:**

L'étude a révélé plusieurs lacunes importantes dans les formations à la recherche sur les politiques et les systèmes de santé. Peu de cours sont proposés en Afrique du Nord, en Amérique latine, en Europe centrale et orientale et au Moyen-Orient. La plupart des cours (116/152) sont dispensés en anglais. Les soutiens institutionnels pour les cours sont rares, et de nombreuses institutions ne disposent pas de la masse critique d'individus suffisamment formés pour accompagner les étudiants pendant et après leur doctorat. La définition des compétences requises pour la recherche sur les politiques et les systèmes de santé manque d'homogénéité d'une institution à une autre. Les collaborations entre disciplines, qui permettent d'offrir toutes les perspectives méthodologiques nécessaires pour traiter ce sujet, restent insuffisantes. Par ailleurs, le manque de solutions alternatives aux cours dispensés sur place peut exclure certains publics étudiants, comme les responsables de l'élaboration des politiques.

**Conclusion:**

La formation à la recherche sur les politiques et les systèmes de santé est cruciale pour améliorer les capacités locales à réaliser des recherches de qualité en la matière. Nous proposons six recommandations pour améliorer le contenu, l'accessibilité et la portée de ces formations. Premièrement, créer une base de données de référence contenant toutes les informations sur les formations. Deuxièmement, créer des réseaux pour appuyer la formation. Troisièmement, définir les compétences requises pour la recherche sur les politiques et les systèmes de santé. Quatrièmement, encourager les collaborations multidisciplinaires. Cinquièmement, étendre la couverture géographique et linguistique des formations. Enfin, envisager des formats d'enseignement alternatifs.

## Resumen

**Objetivo:**

Investigar las características de la formación para la investigación en políticas y sistemas de salud a nivel global e identificar las recomendaciones para lograr mejoras y ampliaciones.

**Métodos:**

Se identificaron instituciones que ofrecían formación para la investigación en políticas y sistemas de salud en todo el mundo. En 2014, se inscribieron participantes de instituciones identificadas para una encuesta en línea sobre las características de las instituciones y los cursos impartidos. Se exploraron los resultados de la encuesta durante entrevistas exhaustivas con informadores clave seleccionados.

**Resultados:**

El estudio identificó numerosas deficiencias importantes con respecto a la formación para la investigación en políticas y sistemas de salud. Había pocos cursos en Europa Central y Oriental, Oriente Medio, el Norte de África y América Latina. La mayoría de los cursos (116/152) se impartían en inglés. El apoyo institucional para los cursos solía ser escaso y muchas instituciones carecían de la cantidad básica de individuos formados necesarios para dar apoyo a los estudiantes de doctorado y posdoctorado. Apenas había coherencia entre las instituciones en cuanto a la definición de las competencias necesarias para la investigación en políticas y sistemas de salud. La colaboración entre las disciplinas para ofrecer la variedad de perspectivas metodológicas necesarias no fue suficiente. Asimismo, la falta de alternativas a la formación in situ puede imposibilitar la asistencia de determinado público estudiantil, como responsables políticos.

**Conclusión:**

La formación para la investigación en políticas y sistemas de salud es importante para mejorar la capacidad local para dirigir una investigación de calidad en este campo. Se ofrecen seis recomendaciones para mejorar el contenido, la accesibilidad y el alcance de la formación. Primero, crear un repositorio de información sobre los cursos. Segundo, establecer redes para dar apoyo a la formación. Tercero, definir las competencias con respecto a la investigación en políticas y sistemas de salud. Cuarto, fomentar la colaboración multidisciplinar. Quinto, ampliar la cobertura geográfica y lingüística de los cursos. Por último, considerar formatos de enseñanza alternativos.

## ملخص

**الغرض:**

استقصاء خصائص التدريب على إعداد أبحاث عن السياسات والنظم الصحية حول العالم وتحديد التوصيات الواجب اتباعها بشأن التحسين والتوسع.

**الطريقة:**

قمنا بتحديد المؤسسات التي تقدم التدريب على إعداد أبحاث عن السياسات والنظم الصحية في جميع أنحاء العالم. وفي عام 2014، قمنا بانتداب بعض المشاركين من المؤسسات التي تم تحديدها للمشاركة في استطلاع للرأي عبر الإنترنت عن خصائص المؤسسات والبرامج التدريبية المقدمة. تم استكشاف نتائج استطلاع الرأي أثناء إجراء مقابلات متعمقة مع مجموعة محددة من المبلّغين الرئيسيين.

**النتائج:**

ساهمت الدراسة في الوقوف على العديد من الفجوات الرئيسية في التدريب على إعداد الأبحاث عن السياسات والنظم الصحية. كان هناك القليل من البرامج التدريبية في وسط وشرق أوروبا، أو الشرق الأوسط، أو شمال أفريقيا، أو أمريكا اللاتينية. وكان يتم تدريس معظم البرامج التدريبية (بواقع 116 دورة من إجمالي 152) باللغة الإنجليزية. وكان الدعم المؤسسي للبرامج التدريبية غائبًا في أغلب الأحوال، كما كانت العديد من المؤسسات تفتقر بدورها إلى العدد المطلوب من الأفراد المُدَرّبين اللازمين لدعم طلاب مرحلة الدكتوراه والمرحلة اللاحقة عليها. كان هناك القليل من التجانس بين المؤسسات في وضع تعريفات للكفاءات المطلوبة لإعداد الأبحاث عن السياسات والنظم الصحية. وكان التعاون في مختلف الاختصاصات لتوفير نطاق وجهات النظر المنهجية اللازمة لهذا الشأن غير كافٍ. وعلاوة على ذلك، قد يؤدي عدم وجود بدائل للتعليم الميداني إلى إثناء بعض الفئات المعينة من المتدربين مثل صنّاع السياسات.

**الاستنتاج:**

إن التدريب في مجال إعداد الأبحاث عن السياسات والنظم الصحية مهم لتحسين القدرات المحلية على إجراء بحث نوعي في هذا المجال. ونحن نقدم ست توصيات لتحسين محتوى التدريب وإتاحته وتيسير سبل الحصول عليه. أولاً، إنشاء مستودع المعلومات المتعلق بالبرامج التدريبية. ثانيًا، تأسيس الشبكات لدعم التدريب. ثالثًا، وضع تعريف للكفاءات في مجال إعداد الأبحاث عن السياسات والنظم الصحية. رابعًا، تشجيع التعاون بين الاختصاصات المتعددة. خامسًا، توسيع نطاق التغطية الجغرافية واللغوية للبرامج التدريبية. وأخيرًا، التفكير في تطبيق صيغ تعليم بديلة.

## 摘要

**目的:**

旨在调查全球范围内卫生政策和体系研究培训的特征，以确定改进和扩展建议。

**方法:**

我们确定了在全球范围内提供卫生政策和体系研究培训的机构。 2014 年，我们针对这些机构及其提供课程的特征开展了一项在线调查，并从这些机构招募了相关人员参与调查。 通过对选定的重要知情人进行深入访问，发掘调查结果。

**结果:**

本调查研究发现了一些卫生政策和体系研究培训中存在的重大差距。 中欧和东欧、中东、北非或者拉丁美洲的课程开设不足。 大多数 (116/152) 课程采用英语授课。 课程常常缺乏制度支持，许多机构缺乏经过培训的教员以指导博士生和博士后学员。 机构间对卫生政策和体系研究培训所需能力的定义不一致。 跨学科合作不足，无法为研究课题提供所需的方法论视角范围。 此外，单一的现场授课方式导致某些学员（例如政策制定者）无法参与。

**结论:**

卫生政策和体系研究培训对于提升当地在该领域开展质量研究的能力方面至关重要。 我们提供了六项建议，以改进培训内容、提高培训可得性，同时扩大培训范围。 首先，创建一个课程信息库。 第二，建立培训支持网络。 第三，明确卫生政策和体系研究的能力要求。 第四，鼓励多学科合作。 第五，扩大课程的地域和语言覆盖面。 最后，考虑其他教学方式。

## Резюме

**Цель:**

Изучить особенности подготовки научных кадров по вопросам политики в области здравоохранения и систем здравоохранения в мире и определить рекомендуемые действия для ее улучшения и расширения охвата.

**Методы:**

Были выявлены учреждения, занимающиеся подготовкой научных кадров по вопросам политики в области здравоохранения и систем здравоохранения в разных странах мира. В 2014 году из выявленных учреждений были набраны участники для прохождения электронного опроса, цель которого заключалась в определении особенностей учреждений и преподаваемых курсов. Результаты опроса были проанализированы в ходе содержательных интервью с отдельными ключевыми информаторами.

**Результаты:**

В результате исследования было выявлено несколько существенных пробелов в подготовке научных кадров по вопросам политики в области здравоохранения и систем здравоохранения. Небольшое количество курсов преподавались в странах Центральной и Восточной Европы, Среднего Востока, Северной Африки или Латинской Америки. Большинство (116 из 152) курсов преподавались на английском языке. Институциональная поддержка курсов часто отсутствовала, и во многих учреждениях наблюдался недостаток обученных лиц, необходимых для эффективного руководства аспирантами и докторантами. Разные учреждения имели разное понимание компетенции, необходимой для проведения исследований, касающихся политики в области здравоохранения и систем здравоохранения. Междисциплинарное сотрудничество для представления разнообразных методологических перспектив, необходимое в этой сфере, проводилось в недостаточном объеме. Кроме того, безальтернативность очного обучения может лишить возможности участия в обучении определенных категорий потенциальных студентов, например лиц, ответственных за разработку политики.

**Вывод:**

Подготовка научных кадров по вопросам политики в области здравоохранения и систем здравоохранения необходима для наращивания местного потенциала, чтобы обеспечить возможность проведения исследований в этой сфере. Авторы приводят шесть рекомендаций, нацеленных на улучшение содержания, доступности и охвата подготовки. Во-первых, необходимо создать архив информации о курсах. Во-вторых, необходимо установить связи между учреждениями для содействия обучению. В-третьих, необходимо дать определение компетенции применительно к исследованиям, касающимся политики в области здравоохранения и систем здравоохранения. В-четвертых, необходимо поощрять междисциплинарное сотрудничество. В-пятых, необходимо расширить географический и языковой охват курсов. Наконец, необходимо предоставить возможность альтернативных форм обучения.

## Introduction

Health policy and systems research has a promising role to play in ensuring the effective implementation of health policy, in strengthening health systems and in improving health outcomes globally.^1^^–^^5^ Although several definitions have been proposed, we adopted the widely accepted definition that health policy and systems research:

“[Seeks] to understand and improve how societies organize themselves in achieving collective health goals, and how different actors interact in the policy and implementation processes to contribute to policy outcomes…[to create] a comprehensive picture of how health systems respond and adapt to health policies, and how health policies can shape – and be shaped by – health systems and the broader determinants of health.”^6^

Health policy and systems research bridges the gap between the generation of knowledge and its application to decision-making in health care.^7^ Consequently, some understanding of the field is now seen as essential for health workforce planning because it equips health-care managers and providers with the skills needed to commission and design health systems research and to incorporate research findings into practice.^8^

The central aim of organizations such as the Alliance for Health Policy and Systems Research and Health Systems Global – a professional society – is to promote health policy and systems research. Since 2010, the biennial Global Symposium in Health Systems Research has raised awareness of gaps in health policy and systems research training in low- and middle-income countries and has encouraged policy-makers to increase training capacity, which has led to the establishment of a thematic working group on teaching and learning health policy and systems research in Health Systems Global.

Health policy and systems research often involves applied research that addresses real-life problems and is intended to inform specific policies or programmes while also contributing to health and societal development.^9^^–^^11^ It is essential to ask the right questions and to choose the most appropriate methods for answering them – methods that may draw on disciplines such as anthropology, epidemiology, economics and systems science.^10^ In addition, conducting health policy and systems research requires considerable knowledge of the way policies are implemented and institutions function. Consequently, establishing the field of health policy and systems research has involved developing a common language and common methods, which has been challenging.^12^

Given these challenges, efforts should be made to increase the ability of researchers and institutions to carry out health policy and systems research and to help them communicate their findings to decision-makers, who must themselves have the ability to request and apply such research.^7^^,^^9^^,^^13^^–^^17^ Moreover, globally relatively few people and organizations have the capacity to commission or advocate for, carry out and use the results of health policy and systems research, particularly in low- and middle-income countries.^18^^–^^21^ The training required by researchers and by those who commission and use health policy and systems research is often highly dependent on the context.

Although training in health policy and systems research is being carried out around the world, little is known about the location or nature of the training provided, which makes it difficult to learn from best practice and to address gaps in training. Moreover, it is not clear which competencies and skills should be developed in training programmes nor which methodological approaches, theoretical frameworks or multidisciplinary perspectives should be taught.^9^^,^^22^^,^^23^ The aim of this study was to describe current health policy and systems research training around the world that has a particular focus on low- and middle-income countries. The institutions delivering training, the purpose of training, key characteristics of the courses being taught, and the measures that could be taken to improve training were all examined.

## Methods

We identified providers of health policy and systems research training that was explicitly relevant to low- and middle-income countries, obtained information on course curricula and teaching and learning modalities and explored gaps in training and their causes to make recommendations for improvement. Data were obtained using a mixed-methods study design that included an online survey and in-depth interviews with key informants, which were intended to add depth and help explain survey responses. The two-part survey was administered in English using the SurveyMonkey platform (SurveyMonkey, Palo Alto, United States of America) between July and September 2014. The first part asked respondents for details about the institutions they were associated with that offered training in health policy and systems research and the second, for details of relevant courses they were personally involved in. Box 1 summarizes the recruitment process for survey participants and Box 2 lists course inclusion criteria. Institutional characteristics included geographical location, the type of organization, the duration of training and the competencies that training aimed to develop among students. Characteristics of individual courses included their objectives, target audiences, course content and teaching modalities. Responses were disaggregated by geographical region, type of institution and national income (i.e. high-income countries and low- and middle-income countries). Open-ended responses were summarized and major themes were identified.

Box 1 **Recruitment of participants, worldwide survey of health policy and systems research training, 2014**

i) The mailing lists of organizations and networks involved in health policy and systems research training were obtained by desk research. Then, emails were sent in English to members of these bodies, which included Health Systems Global and its thematic working groups, the Consortium for Health Policy and Systems Analysis in Africa, the Alliance for Health Policy and Systems Research, Afro-Nets, Health Space Asia, the Health Systems Research India Initiative and the European Observatory on Health Systems and Policies.

ii) Snowball sampling, in which email recipients and survey respondents were asked to provide the contact details of others involved in teaching, was used to identify more than 100 additional, potential, survey respondents.

iii) Well-known experts who were running health policy and systems research training programmes were consulted to ensure no important courses were missed. Over 120 follow-up emails were sent to potential, survey respondents in under-represented regions to boost participation in these regions.

iv) An online search for relevant courses was conducted via Google (Google, Mountain View, United States of America) using the following search strategy: [[“health policy” OR “health systems” OR “HSR” OR “HPR” OR “health policy and planning”] AND [“course” OR “module” OR “workshop” OR “seminar” OR “class” OR “lecture” OR “short course”] AND “research”]. In addition, a search for relevant courses at schools of public health worldwide was carried out. Potential survey respondents identified in these searches were contacted directly.

Box 2 **Course inclusion criteria, worldwide survey of health policy and systems research training, 2014**

A course was included in the survey if the following three criteria were satisfied:

i) it was a course, seminar, practicum or other recognized form of education that included health policy and systems research or a related term (e.g. health systems or services research, health policy research or implementation research) in its title, objectives or description or it was a course or programme that explicitly included a health policy and systems research component;

ii) it included the teaching of research methods or the critical appraisal of research methods;

iii) the course title, objectives or description made clear it was relevant to low- and middle-income countries, irrespective of the income level of the country in which the institution was located.

We selected 29 key informants across all regions of the world from among survey respondents who were willing to be interviewed. All had reported involvement in two or more courses relevant to health policy and systems research in their survey responses, had been recommended by two or more survey participants as key institutional contacts in the field and had a long track record of carrying out, publishing and teaching health policy and systems research. We used survey responses to identify issues that required further examination: we explored any unusual answers given by participants and obtained more information on themes mentioned frequently in responses to open-ended questions. Subsequently, interview transcripts were analysed thematically using NVivo 10 (QRS International, Melbourne, Australia). Finally, we selected representative quotations that illustrated themes on which there was a high degree of agreement between participants and which may, therefore, be transferable to a variety of settings.

The survey was exempted from the need for ethical approval by the ethics committee of the London School of Hygiene & Tropical Medicine and participants were informed that consent was implicit in their participation. The qualitative part of the study received ethical approval (No. 8485) and informed consent was sought from all participants. All transcripts were anonymized and treated as confidential.

## Results

**Fig. 1:**
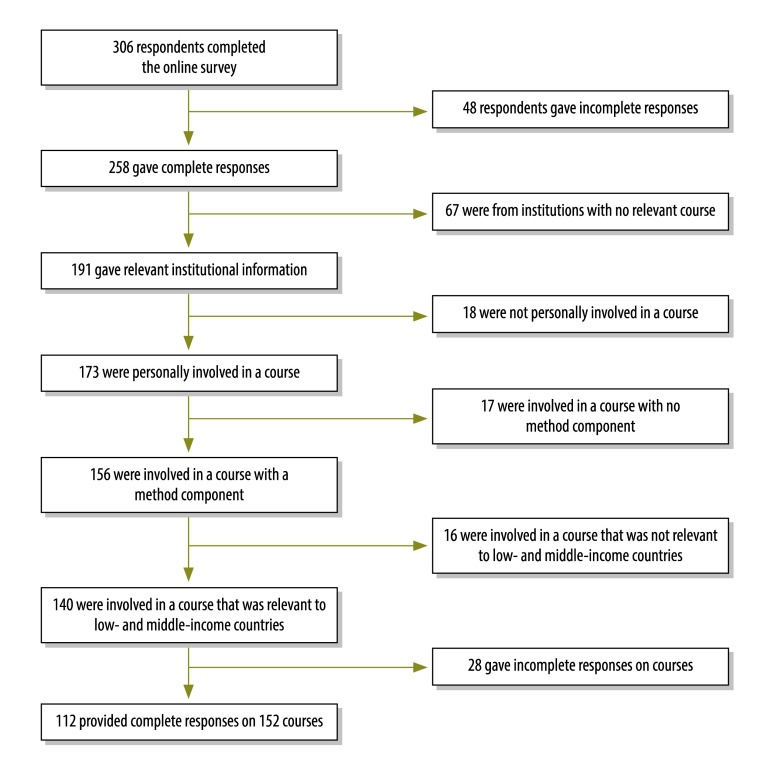
Recruitment of participants, worldwide survey of health policy and systems research training, 2014

In total, 306 respondents completed the online survey, 191 of whom provided information on 169 different institutions they were associated with that offered health policy and systems research courses. Of the 191, 140 reported on one or more courses that met the inclusion criteria (Box 2). After removing incomplete entries, we analysed survey responses from 112 individuals, from different institutions, who provided information on 152 courses they were directly involved with (Fig. 1).

### Geographical distribution

**Fig. 2:**
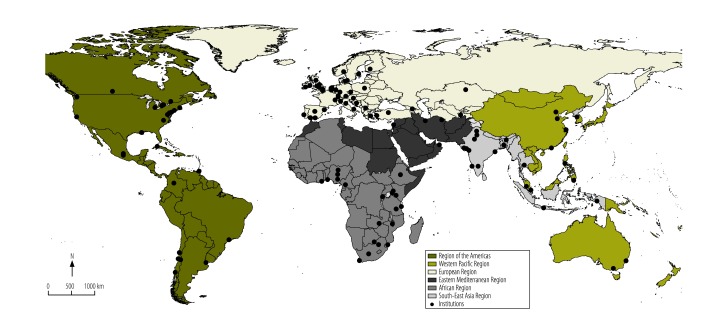
Institutions offering health policy and systems research training, by World Health Organization region, 2014

Institutions teaching health policy and systems research were found in every World Health Organization region (Fig. 2). The highest proportion of institutions was in the European Region (42%; 71), predominately in western Europe. In addition, 17% (29) of institutions were in the Region of the Americas, 16% (27) were in the African Region, 15% (25) were in the South-East Asia Region and 8% (14) were in the Western Pacific Region but only 2% (3) were in the Eastern Mediterranean Region. Few institutions offered relevant training in central and eastern Europe, the Middle East or Latin America and there weres none in North Africa.

### Instruction language

The primary language of instruction was English: 76% (116) of courses were offered only in English, 8% (12) were offered in both English and another language and 16% (24) were offered only in a language other than English.

### Gaps in training

#### Institutional factors

Institutional support was found to be critical for developing health policy and systems research training. Many key informants said they felt isolated and were not fully integrated into their institution’s infrastructure. It was also difficult to obtain funding for the development of new courses and there were few individuals able to provide mentorship and support for doctoral and postdoctoral students. A key informant at a research institution in a low- or middle-income country said,

“It’s particularly difficult to get programmatic-type funding so that you can really build a set of people … in terms of sustainable careers, health systems research is not part of the fabric of academic institutions here. So universities really don’t have those kinds of people yet. And they’re not likely to get them I would say for quite some time unless there are profitable ways to run master’s courses.”

It was also felt that existing institutional structures and processes did not encourage multidisciplinary collaboration on the curriculum and that courses related to health policy and systems research were often delivered with little integration between departments. A more fundamental issue was the perception that health policy and systems research is a new field that may lack legitimacy, both within academic institutions and among decision-makers. In addition, informants thought the motivation and demand for health policy and systems research teaching and training were different from those for other disciplines because health policy and systems research is used in decision-making. One respondent at a research institution in a low- or middle-income country commented, “I think one of the bits that is missing is recognition from government that these kinds of areas are important.”

Many institutions did not specifically formulate the core competencies required for health policy and systems research. From the survey, of 191 respondents from institutions offering training, only 110 (58%), from 92 different institutions, gave details of competencies that were specific to study programmes offering health policy and systems research training. Moreover, 13% (14) of the 110 were not able to specify distinct competencies and 11% (12) gave the same information when asked about programme competencies and specific learning objectives for courses. When distinct health policy and systems research competencies were described, they were very broad and often not articulated as competencies: for example, the capability to apply or use a set of related knowledge, skills and abilities to successfully perform in a defined work setting. Moreover, the most frequently reported competency – “to gain a general background in public health” – is not considered an educational competency.^24^ Other reported competencies were broadly related to applying health policy and systems research skills in a work setting: for example, the ability to: (i) conduct policy analysis; (ii) develop and use leadership skills; (iii) develop and use health financing skills; and (iv) conduct analyses based on research questions.

#### Student audiences

**Fig. 3:**
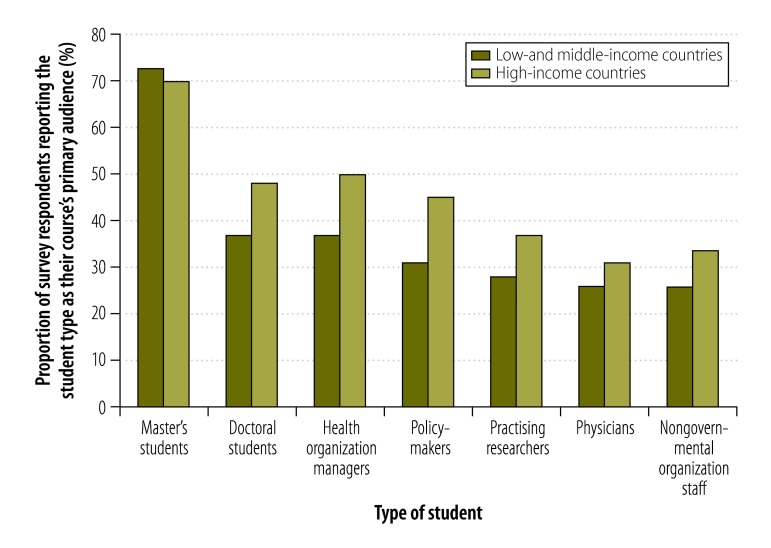
Student audiences, health policy and systems research courses, worldwide, 2014

Note: Because survey respondents were permitted to name more than one primary student audience, the totals for the country groups exceeded 100%.

According to survey respondents, almost 70% (106) of health policy and systems research courses were embedded in a bachelor’s, master’s or doctoral degree programme or another diploma programme, and the largest target audience were master’s students (Fig. 3). However, key informants suggested that many of these master’s students were probably also health organization managers, policy-makers or similar professionals. Key informants felt strongly that certain potential students, such as staff working in health care, policy-makers or administrators, who could benefit from training were not being reached. More flexible teaching models could be helpful. A key informant from a university who worked for a nongovernmental organization in a low- or middle-income country said,

“If we can get more and more people who are in the policy-making processes and people who are really policy implementers, people from civil society groups … I think certainly we would make much more impact.”

#### Course content

**Fig. 4:**
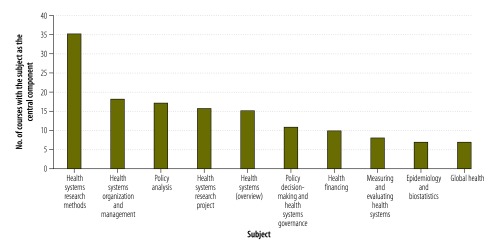
Most commonly taught subjects, health policy and systems research courses, worldwide, 2014

Note: The total number of courses included in the survey was 152.

Overall, 72% (109) of health policy and systems research courses taught at least one quantitative research method, 76% (116) taught at least one qualitative research method and 88% (134) taught at least one of either method. As shown in Fig. 4, the course content was highly varied, as was confirmed by key informants. However, it often lacked some fundamental elements. Thus, many respondents suggested that a basic understanding of health systems and systems thinking would be particularly beneficial for students – the teaching of frameworks was frequently mentioned. A respondent at a university in a high-income country said,

“Getting people to come to a common ground on a framework of concepts and definitions and approaches to health systems seems to be a real prerequisite, because then you can start using that as a way to reach into the system as a way to talk about it, understand it, appreciate the complexity and the interrelationships of the components.”

Health policy and systems research teaching appeared to depend on courses that focused on methods in general and that were not restricted to health policy and systems research applications, possibly because many organizations did not have the critical mass of teachers and students needed to provide specific courses in this field.

#### Teaching formats

Overall, 80% (122) of courses were provided only on-site at an institution, 12% (18) were offered only online and 8% (12) were offered both on-site and online. Most key informants were interested in offering more online and blended learning (i.e. teaching with both on-site and online components) because they felt that such courses might be more accessible. However, the lack of time and resources to develop online materials was a major barrier to offering flexible teaching formats. A respondent at a university in a low- and middle-income country commented,

“I think the question [is] how we discover a balanced approach where you deliver some content online and at the same time you allow for that interactive, participative process, because people learn from each other as well, particularly when you have students sitting in the same room.”

**Fig. 5:**
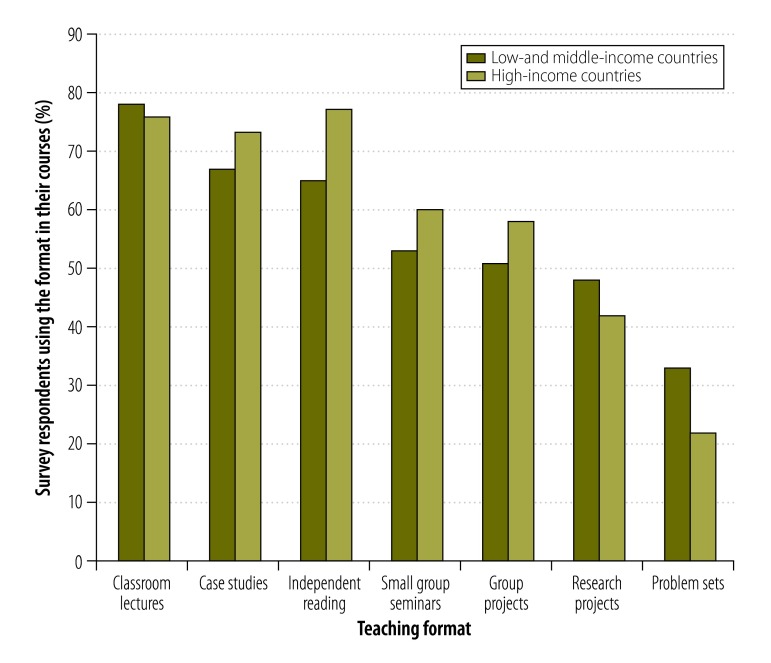
Common teaching formats, health policy and systems research courses, worldwide, 2014

Fig. 5 shows the most common teaching formats reported. The majority of courses used traditional teaching methods in an academic setting but key informants emphasized the importance of drawing on real-life examples in teaching. Teaching approaches regarded as valuable by key informants included: (i) giving students practical experience in the field; (ii) enabling students to identify their own research questions or projects; (iii) enabling students to practise engaging with policy-makers, for example, through simulations or by role-playing; and (iv) helping students to understand how health policy and systems research can inform health policy and practice. A respondent at a university who worked for a nongovernmental organization in a low- or middle-income country said,

“Going to the field and actually seeing how a health system works is more important. Or doing some real health systems work. Understanding the drug market, how it is produced and distributed – those sorts of things are very useful. Or just going to a hospital or primary centre and seeing how it actually functions in real life and what are the constraints.”

## Discussion

This global assessment of current health policy and systems research training identified several gaps in training capacity. First, disproportionately few institutions offered training in central and eastern Europe, the Middle East or Latin America, with none in North Africa. Second, English was the predominant primary language of course instruction. Third, there was often a lack of support for courses from parent institutions. Fourth, many institutions did not have the critical mass of trained individuals needed to support doctoral and postdoctoral students. Fifth, there was a lack of consistency between institutions in definitions of the competencies required for health policy and systems research. Sixth, there was insufficient collaboration across disciplines to provide the range of methodological perspectives required by health policy and systems research. Finally, there was a lack of alternatives to on-site teaching for degree programmes, which may preclude participation by specific student audiences, such as policy-makers and frontline health workers. The broad, question-driven nature of health policy and systems research makes it exceptionally valuable for understanding and evaluating complex health systems issues. However, there is a risk that the subject becomes amorphous and challenging to teach. Efforts should continue to define and develop the field and strengthen the training and mentorship capacity of global networks, institutions and individuals.

The main limitation of this study was that both recruitment emails and the survey were in English; moreover, key informants were selected from survey respondents. The reliance on circulating emails through networks that were largely English-speaking may have led to fewer courses being identified in regions where English is not the first language. In addition, we may have missed relevant training courses that could not be located online or that were not well known to the health systems research community. Finally, the gaps in training we identified were based on the instructors’ perspective – the student’s viewpoint was not represented.

Despite these limitations and the caution required in interpreting the study’s findings, several points of agreement emerged, which led to six recommendations for action. First, create a repository of information on health policy and systems research courses. Key informants expressed an interest in having access to an updated course repository that included learning objectives and an overview of content – such a repository can be accessed through the corresponding author on request. Sharing open-access learning materials, curricula and syllabi wherever possible would help individuals develop, update or adapt their own courses and enable them to learn from best practice in centres of excellence or in countries with similar problems. Second, expand networks that support health policy and systems research training and offer opportunities for mentorship and sharing knowledge. Identifying individuals and institutions with similar interests is an important first step in creating such networks,^25^ especially since the ability to interact with, and learn from, other instructors and their experience is critical for developing training. The lack of doctoral and postdoctoral students engaged in health policy and systems research in low- and middle-income countries has been noted elsewhere.^13^^,^^26^ Expanding networks that can link students with potential mentors, both within and between institutions, would help support learners. Furthermore, networking across disciplines and institutions may help transcend barriers to training associated with weaknesses in particular institutions by capitalizing on the strengths of others.^23^ Third, define competencies in health policy and systems research and an approach to adapting them to diverse contexts and audiences. Defining competencies would help establish standards for the knowledge, skills and abilities needed in the field and adapting them will ensure that they are relevant. Moreover, it would help existing and emergent health policy and systems research communities engage in a dialogue about competencies and help refine them over time – this could also involve policy-makers and other individuals who request or use the information obtained through health policy and systems research. Fourth, encourage multidisciplinary collaboration. Health policy and systems research encompasses a diversity of skills and requires an ability to work across disciplines. Overcoming institutional and professional boundaries and expectations is central to promoting coherent training programmes. Fifth, expand the geographical and language coverage of courses. Although possibly an artefact of sampling, the observed apparent under-representation of health policy and systems research training in particular regions highlights the need to diversify predominantly Anglophone training. Courses and resources should be developed in languages other than English and those already available should be shared more widely. In under-represented regions, engagement with financial donors is essential for developing training capacity. Sixth, consider alternative teaching formats for courses. Providing health policy and systems research courses within dedicated university-based programmes offers clear benefits and should continue: such courses help develop well rounded researchers with a broad knowledge and a range of capabilities.^22^^,^^27^ However, since these courses may preclude some student audiences, other formats should be considered, such as blended learning, which includes both on-site and online components, and short and part-time courses. Given the increasing need for flexible training, it is also important to learn about innovation in teaching approaches and to identify features that are transferrable between settings.

In conclusion, health policy and systems research is an important field of research of increasing international interest. It has the potential to promote new and more rigorous research and greater use of research findings, which can strengthen health systems and improve population health. Consequently, high-quality, responsive training is an important vehicle to accelerate this process. Our six recommendations for action could help improve the content, accessibility and reach of training in the field.
